# Ca-Doping Cobalt-Free Double Perovskite Oxide as a Cathode Material for Intermediate-Temperature Solid Oxide Fuel Cell

**DOI:** 10.3390/molecules29132991

**Published:** 2024-06-23

**Authors:** Liangmei Xue, Songbo Li, Shengli An, Qiming Guo, Mengxin Li, Ning Li

**Affiliations:** 1 School of Chemistry and Chemical Engineering, Inner Mongolia University of Science and Technology, Baotou 014010, China; 15941277965@163.com (L.X.); g18347114572@hotmail.com (Q.G.); lmx3210@hotmail.com (M.L.); ln19981213@outlook.com (N.L.); 2 School of Materials and Metallurgy, Inner Mongolia University of Science and Technology, Baotou 014010, China; san@imust.edu.cn

**Keywords:** IT-SOFC, Fe-based, Ca doping, electrochemical performance

## Abstract

Mixed oxygen ion and electron-conducting materials are viable cathodes for solid oxide fuel cells due to their excellent oxygen transport kinetics and mixed electrical conductivity, which ensure highly efficient operation at low and medium temperatures. However, iron-based double perovskite oxides usually exhibit poor electrocatalytic activity due to low electron and oxygen ion conductivity. In this paper, Ca is doped in PrBaFe_2_O_5+δ_ A-site to improve the electrochemical performance of PrBaFe_2_O_5+δ_. Results show that replacing Pr with Ca does not change the crystal structure, and the Ca doping effectively increases the adsorbed oxygen content and accelerates the migration and diffusion rate of O^2−^ to the electrolyte|cathode interface. The polarization resistance of the symmetric cell PC_0.15_BF|CGO|PC_0.15_BF is 0.033 Ω·cm^2^ at 800 °C, which is about 56% lower than that of PBF, confirming the enhancement of the mixed conduction of oxygen ions and electrons. In addition, the anode-supported single cell has a peak power density of 512 mW·cm^−2^ at 800 °C.

## 1. Introduction

With the increase in energy consumption, the demand for energy conversion technology and equipment has become more urgent. Solid oxide fuel cell (SOFC) is one of the most promising energy conversion devices [1,2] that can directly convert chemical energy into electricity with a conversion efficiency of 60–65%. The high operating temperature (800–1000 °C) of traditional SOFCs leads to problems such as short life and high cost, which limit the commercialization of SOFC technology [3,4]. However, when the operating temperature of SOFC is reduced to medium and low temperatures, the cell output performance rapidly declines mainly due to the rapid increase in polarization resistance on the cathode side, resulting in poor oxygen reduction reaction (ORR) dynamics [5,6]. Therefore, the development of cathode materials with excellent electrochemical properties and low cost is a current research hotspot.

In recent years, the mixed oxygen ion and electronic conductor has attracted extensive attention from researchers due to its excellent oxygen transport kinetics and mixed conductivity, which can diffuse the reactive active site from the narrow three-phase interface to the entire electrode [7]. LnBaCo_2_O_5+δ_ (Ln is a rare earth element) double perovskite oxides have been widely studied as SOFC cathode materials due to their excellent electrical conductivity and oxygen transport properties [8,9]. In the LnBaCo_2_O_5+δ_ structure, the LnO and BaO layers are alternately arranged as -BaO-CoO_2_-LnO_δ_-CoO_2_-BaO along the C-axis. This layered and ordered structure reduces the binding energy of the Ln-O bond; oxygen vacancies are located in the LnO_δ_ layer, generating independent oxygen transport channels and promoting the rapid diffusion of O^2−^ [10,11]. This unique arrangement of oxygen vacancies makes LnBaCo_2_O_5+δ_ exhibit high oxygen vacancy concentration, excellent oxygen surface diffusion coefficient, and oxygen surface exchange capacity; therefore, it has good electrocatalytic activity. However, LnBaCo_2_O_5+δ_ generally has a high average thermal expansion coefficient (TEC) and poor thermal matching with electrolyte materials. On the one hand, the change of the Co^3+^ spin state with the increase in temperature results in the physical expansion of the octahedron CoO_6_. On the other hand, the reduction of Co causes the lattice oxygen defect to lead to lattice chemical expansion, which intensifies LnBaCo_2_O_5+δ_ lattice shrinkage. The cathode easily falls off and affects the output performance after a long time, which is also a major shortcoming of Co-based perovskite materials [12]. The most direct way to overcome this factor is to reduce the Co content through B-site metal ion doping, thereby reducing the TEC of the material. Kim et al. [13] studied the effect of Fe doping on the electrochemical performance of LnBaCo_2-x_Fe_x_O_5+δ_ (Ln=Nd and Gd) double perovskite cathodes and found that Fe doping reduced the average TEC due to the formation of a strong Fe-O bond by the introduced Fe ions, which inhibited the generation of oxygen vacancy. The chemical expansion caused by the lattice expansion was then controlled, and the spin change of Co from low to high was also inhibited by the substitution of high spin Fe^3+/4+^, which reduced the physical expansion of the material. Yoo et al. [14] studied Fe substitution for Co in PrBaCo_2_O_5+δ_ materials and found that proper Fe doping can improve the structural symmetry and the stability of the materials under the REDOX atmosphere. Compared with Co-based cathode materials, Fe-based cathode materials have lower TEC, good chemical stability, and low cost. However, due to the low covalency of Fe^4+^-O bonds compared with Co^4+^-O bonds, electron localization increases, reducing electrical conductivity. Therefore, the performance of this material needs to be optimized further for commercialization. Doping is an effective method for improving the electrochemical catalytic activity of LnBaFe_2_O_5+δ_ peroxides. Some studies used alkaline earth metal ions doped in the A-site of double perovskite to improve the structural stability and electrochemical performance of the material. Wang [15] et al. studied the effect of Ca doping on the physicochemical properties and electrochemical performance of GdBaFe_2_O_5+δ_ material. The addition of Ca increased the electron hole and oxygen content of GdBaFe_2_O_5+δ_, thereby increasing its conductivity and decreasing its polarization resistance. Jin [16] et al. prepared Ca-doped PrBaCoFeO_5+δ_, and the results showed the addition of Ca improved the oxygen catalytic activity and phase structure stability while reducing TEC and production cost.

Although the introduction of Ca and Fe reduces the TEC of PrBaCo_2_O_5+δ_, the TEC is still higher than that of common electrolyte materials. LaBaFe_2_O_6-δ_ oxides have an A-site cation disordered perovskite structure, which is a kind of mixed oxygen ionic and electronic conductor, of which PrBaFe_2_O_5+δ_ (PBF) is mainly investigated as an electrocatalytic catalyst material for the oxygen-reduction reaction. In this work, Pr_1–x_Ca_x_BaFe_2_O_5+δ_ (x = 0, 0.05, 0.1, 0.15, 0.2, PC_x_BF) series of materials were prepared by replacing Pr with Ca at the A-site to enhance its catalytic activity as a cathode. The physical phase structure, conductivity, and electrochemical properties were investigated. With the lattice expansion of Ca doping, the oxygen vacancy concentration increases, which promotes oxygen migration and the redox reaction process. Therefore, the single cell prepared with PC_x_BF as the cathode shows good electrochemical performance, indicating that PC_x_BF has application potential as an intermediate-temperature solid oxide fuel cell (IT-SOFC) cathode material.

## 2. Results and Discussion

### 2.1. Elemental Composition and Crystal Structure

**Figure 1:**
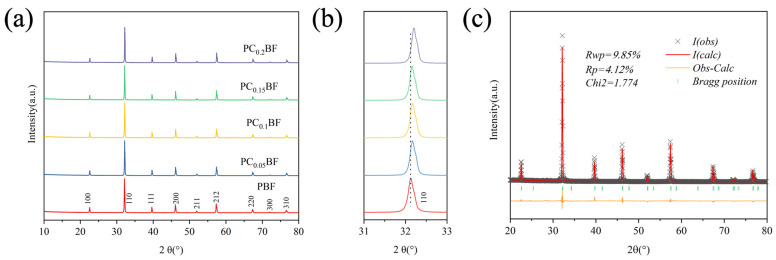
(**a**) XRD patterns of PC_x_BF; (**b**) local magnification; (**c**) Rietveld refined XRD pattern of PC_0.15_BF.

Figure 1a shows the XRD patterns of the sample PC_x_BF (x = 0, 0.05, 0.1, 0.15, 0.2) obtained after calcination at 1100 °C in the air for 5 h. PC_x_BF is a pure phase-layered double-perovskite structure [17]. The local region of the XRD pattern is enlarged, as shown in Figure 1b. The diffraction peak of PC_x_BF gradually shifts to a large angle with the increase of the Ca doping amount, indicating that the cell volume of PC_x_BF gradually decreases and the lattice shrinks with the increase in the doping amount. Because the radius of Ca^2+^ (1.34 Å) is similar to that of Pr^3+^ (1.30 Å) [18], the lattice shrinkage is due to the increased Pr^4+^ and Fe^4+^ content. To study the effect of Ca^2+^ doping on the crystal structure of PrBaFe_2_O_5+δ_ further, the XRD pattern of PC_x_BF was refined by using the Rietveld method (Figure 1c and Figure S1). The refined pattern agreed with the XRD pattern. Table S1 shows the results of the refined pattern. PC_x_BF is a layered double-perovskite structure with a P4/mmm space group. Ca doping does not change the original crystal structure, but the cell volume gradually decreases with the increase in the doping amount, which is consistent with the results of diffraction peak high-angle shift. The (110) peak of layered double perovskites with space group P4/mmm is usually composed of two sharp diffraction peaks. However, in PC_x_BF, these two peaks combine into a wider diffraction peak, indicating that PC_x_BF has good structural symmetry. Similar phenomena have also been reported in the literature [19,20].

**Figure 2:**
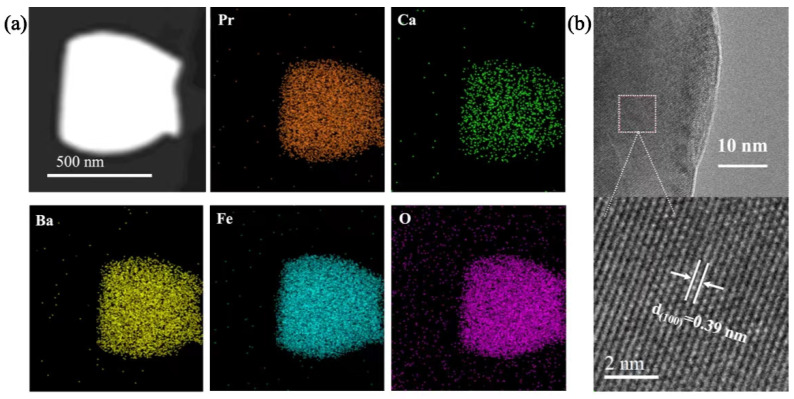
(**a**) EDS images of PC_0.15_BF; (**b**) HR-TEM images of PC_0.15_BF.

Figure 2a shows the EDS spectrum of PC_0.15_BF powder. Pr, Ca, Ba, Fe, and O are evenly distributed, and no element aggregation is found, confirming that Ca successfully replaces Pr at the A position. The sample PC_0.15_BF was characterized by high-resolution TEM, as shown in Figure 2b. The calculated lattice spacing of 3.9 Å corresponds to the (100) crystal plane. To study the chemical stability between the cathode material and electrolyte, electrolyte powder (CGO) was mixed with cathode material PC_0.15_BF at an equal mass ratio, and an XRD test was conducted after holding at 1100 °C for 5 h. The results are shown in Figure S2. Except for the diffraction peaks of PC_0.15_BF and CGO electrolyte powders, no other diffraction peaks appear, indicating no evident solid phase reaction between PC_0.15_BF and CGO electrolytes that have good chemical compatibility.

### 2.2. X-ray Photoelectron Spectroscopy Analysis

**Figure 3:**
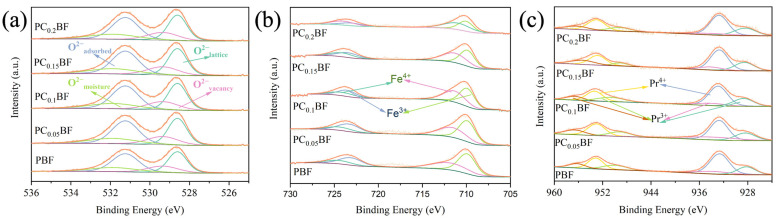
XPS curves of PC_x_BF: (**a**) O1s; (**b**) Fe2p; (**c**) Pr3d.

To study the effect of Ca doping on materials, the surface ion valence states of PC_x_BF series materials were analyzed using XPS. Because the oxygen in the peroxide lattice plays an important role in the ORR of the cathode material, the results of peak-splitting fitting of the O1s peak in the PC_x_BF material from the literature are shown in Figure 3a [21]. The O1s spectrum at the binding energy of 526–535 eV is divided into water oxygen (O_moisture_), absorbed oxygen (O_adsorbed_), vacancy oxygen (O_vacancy_), and lattice oxygen (O_lattice_). The values of (O_adsorbed_ + O_vacancy_)/O_lattice_ were calculated and are listed in Table 1. The ratio gradually increases with the increase in the doping amount, indicating the doping of Ca^2+^ at the A position increases the oxygen adsorption capacity, and more oxygen vacancies can be obtained, which is consistent with the calculated results of oxygen content (Table S2). Figure 3b,c shows the XPS spectra and fitting curves of PC_x_BF Fe2p_3/2_ and Pr3d at room temperature, respectively. The main binding energy peaks of 932.2 eV (±0.2 eV) and 952.7 eV (±0.2 eV) belong to Pr^4+^3d_5/2_ and Pr^4+^3d_3/2_, respectively. The absorption peaks at 928.0 eV (±0.2 eV)/933.6 eV (±0.21 eV) and 949.0 eV (±0.2 eV)/956.3 eV (±0.2 eV) can be attributed to Pr^3+^3d_5/2_ and Pr^3+^3d_3/2_, respectively [22,23,24]. The main peaks of binding energy at 709.3 eV (±0.16 eV) and 722.8 eV (±0.16 eV) belong to Fe^3+^2p_3/2_ and Fe^3+^2p_1/2_, respectively [25,26]. The main binding energy peaks of 710.8 eV (±0.18 eV) and 723.7 eV (±0.2 eV) are attributed to Fe^4+^ 2P_3/4_ and Fe^4+^2p_1/2_, respectively. The percentage values of Pr^4+^/^3+^ and Fe^4+/3+^ are shown in Table 1. With the increase in Ca^2+^ doping, the valence state of Pr gradually increases, leading to lattice shrinkage, and the proportion of Fe^4+^ gradually increases, indicating the transition metal element (Fe) in the double perovskite oxide is oxidized to a higher valence state when Ca replaces Pr.

### 2.3. Electrical Conductivity

**Figure 4:**
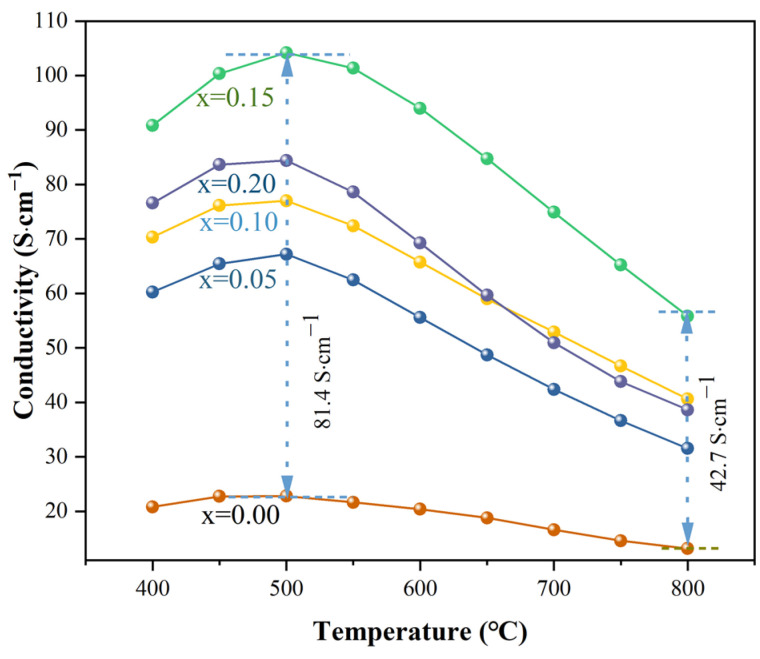
Conductivity of PC_x_BF in air atmosphere.

Figure 4 shows the change curves of the conductivity (σ) of PC_x_BF (x = 0, 0.05, 0.1, 0.15, 0.2) at different temperatures in a dry air atmosphere. In the REDOX atmosphere, because the ionic conductivity is two orders of magnitude lower than the electronic conductivity, the conductivity is equated with the electronic conductivity in this paper [27]. Figure 4 shows that when the temperature is below 500 °C, the conductivity of PC_x_BF increases with the increase in temperature. This process follows the mechanism of small polaron transition, and thermal excitation accelerates the carrier migration rate and increases electronic conductivity [20]. When the temperature is higher than 500 °C, the conductivity decreases with the increase in temperature, which is manifested as the metal conductive behavior, because the amount of lattice oxygen escape increases substantially with the increase in temperature, and the oxygen vacancy concentration also increases. To maintain electrical neutrality, part of the Fe^4+^ is reduced to Fe^3+^, which lowers the concentration of carriers and reduces electronic conductivity. The conductivity of PC_x_BF increases with the increase in Ca doping. When x = 0.15 and T = 500 °C, σ reaches a peak value of 104.18 S·cm^−1^, which meets the requirement that the conductivity of cathode material should be greater than 100 S·cm^−1^ [28]. In perovskite cathode materials, charge carriers are conducted by B-O-B network structure, doped in cations with lower valence states. To maintain the charge neutrality of PBF, the valence of B-site cations increases. Therefore, with the addition of Ca, the number of Fe^4+^ ions increases, and B^3+^-O-B^4+^ small polarons increase. At the same time, the introduction of Ca reduces the average valency of transition metal ions in the material, resulting in the formation of oxygen vacancies. Oxygen vacancies in the lattice have high mobility and participate in conduction, resulting in an increase in conductivity. With the increase of the doping amount, oxygen content decreases and numerous oxygen vacancies are formed; an extremely high oxygen vacancy concentration hinders electron conduction and reduces electron conductivity [29]. Therefore, the electrical conductivity of the material decreases with the increase in Ca.

### 2.4. Thermal Expansion Behavior

**Figure 5:**
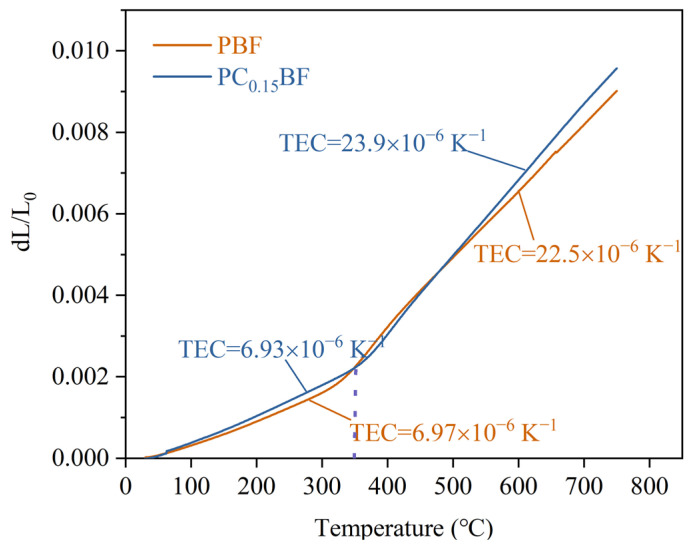
Thermal expansion curve of PC_x_BF (x = 0, 0.15) at air atmosphere.

To prevent the rupture and degradation of the cell caused by heat stress at high temperatures, the TEC of the cell components should be closed. Figure 5 shows the thermal expansion curves of PC_x_BF (x = 0, 0.15) samples in the range of 30–750 °C under air atmosphere. At a given temperature, the thermal expansion of the sample (*dL*/*L*_0_) increases with the increase in Ca content, which is because of the lattice shrink caused by Ca replacing Pr at the A-position, and the increase in oxygen loss leads to the increase in TEC. In addition, the TEC of the material increases with the increase in temperature, which is caused by the escape of a large amount of lattice oxygen [30]. In the temperature range of 30–750 °C, the average TEC of PC_0.15_BF is 13.3 × 10^−6^ K^−1^, which is considerably lower than that of PrBaCo_2_O_5+δ_ (23.4 × 10^−6^ K^−1^) because the bond energy of Fe-O is stronger than that of Co-O [24]. This outcome indicates that the substitution of Co with Fe in PrBaCo_2_O_5+δ_ can effectively improve thermal matching between PrBaCo_2_O_5+δ_ and electrolyte CGO.

### 2.5. ORR Activity

**Figure 6:**
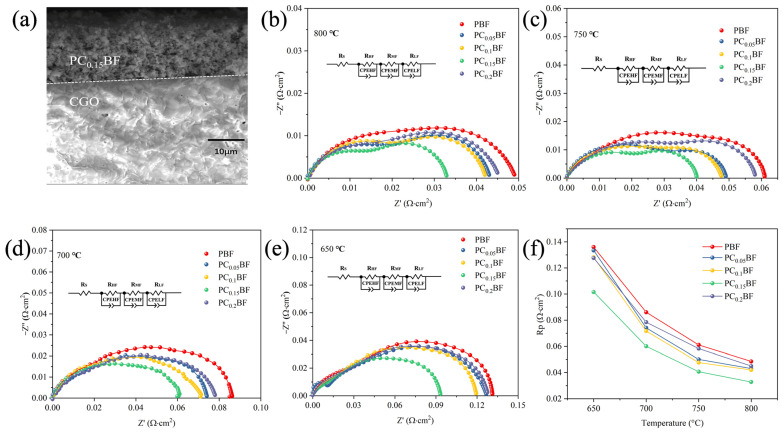
ORR performance of symmetrical cells at different temperatures: (**a**) SEM image of the PC_0.15_BF|CGO cross-section; (**b**–**f**) Arrhenius plots of Rp values of PC_x_BF in the temperature range of 650–800 °C.

To study the electrochemical performance of PC_x_BF series cathode materials, the AC impedance spectrum of PC_x_BF|CGO|PC_x_BF symmetrical cell supported by electrolyte was tested in an air atmosphere. Figure 6a shows the SEM cross-section image of PC_0.15_BF|CGO after the symmetrical cell test. The PC_0.15_BF cathode shows satisfactory continuous contact with the dense CGO substrate, where the electrolyte thickness is about 350 μm. Figure 6b–e shows the AC impedance spectrum of symmetrical cell PC_x_BF|CGO|PC_x_BF at 800–650 °C. *R_s_* (*R_HF_*//CPEHF *R_MF_*//CPEMF *R_LF_*//CPELF) equivalent circuit diagram is used to fit the measured AC impedance spectrum [28,31], where *R_s_* stands for ohmic resistance and *R_HF_*, *R_MF_*, and *R_LF_* correspond to high-, medium-, and low-frequency polarization resistance, respectively. The total resistance is *R_p_* (*R_p_ = R_HF_ + R_MF_ + R_LF_*). *R_o_* is defined as the arc cut at high frequency and is often zeroed out for convenience. Figure 6f is the polarization resistance value obtained after fitting. When the test temperature is constant, the doping amount of Ca continues to increase, and the polarization resistance *R_p_* shows a trend of first decreasing and then increasing, which is because Ca doping effectively increases the adsorbed oxygen content at position A, and the increase in oxygen vacancy concentration promotes O^2−^ transfer in the cathode and improves the oxygen reduction reactivity of the PC_x_BF|CGO interface, thus reducing polarization resistance. The polarization resistance value of PC_0.15_BF at 800 °C is 0.033 Ω·cm^2^, which is much lower than that of PBF. It is also lower than that of other Fe-based cathode materials reported in the literature (Table 2), which indicates that PC_0.15_BF has great development potential as a cathode material. However, with the continuous increase of the doping amount, excessive oxygen vacancy concentration is prone to defect association, resulting in the localization of oxygen vacancy [13], which reduces the transmission rate of oxygen ions and further increases *R_p_*.

**Figure 7:**
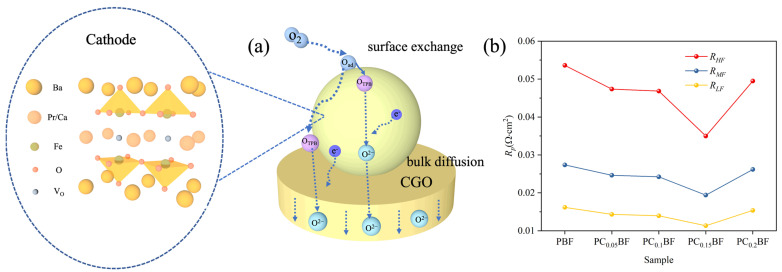
(**a**) Diagram of the ORR mechanism in the cathode; (**b**) polarization resistance values at 800 °C.

The electrochemical reduction reaction of oxygen on the electrode is a complex process, as shown in Figure 7a. The overlapping steps mainly include the adsorption and deionization of O_2_ on the cathode surface, the combination of O atom and e^−^ to form O^2−^ (which occurs at the three-phase interface), and the transfer of O^2−^ between the electrode and the electrolyte. To clarify further the specific role of Ca doping in these steps, Figure 7b shows the different frequency segments of PC_x_BF|CGO|PC_x_BF at 800 °C, namely, *R_p_* values corresponding to high frequency (HF, transfer of oxygen ions from TPB to electrolyte), medium frequency (IF, charge transfer), and low frequency (LF, adsorption and dissociation of oxygen). Compared with PBF, *R_p_* values in the high- and low-frequency regions of PC_x_BF decrease considerably, indicating the proper amount of Ca doping accelerates oxygen reduction. In addition, R_HF_ > R_MF_ > R_LF_ indicates the transfer of oxygen ions is a rate control step in ORR [32].

**Table 2:** Polarization resistance of Fe-based materials (Ω·cm^2^).

Sample	Electrode	Temperature (°C)	*R_p_* (Ω·cm^2^)	Reference
La_0.6_Sr_0.4_Fe_0.8_Cu_0.2_O_3-δ_	SDC	800	0.07	[33]
Bi_0.5_Sr_0.5_Fe_0.95_P_0.05_O_3−*δ*_	GDC	700	0.18	[34]
La_0.5_Sr_0.5_Fe_0.9_Mo_0.1_O_3–δ_	SDC	700	0.211	[35]
Sr_0.95_Ti_0.3_Fe_0.6_Ni_0.1_O_3−δ_	GDC	800	0.06	[36]
Sm_0.8_La_0.2_BaFe_2_O_6-δ_	SDC	800	0.12	[37]
Pr_1.85_Ca_0.15_BaFe_2_O_5+δ_	CGO	800	0.033	This Work

### 2.6. Single-Cell Performance Test

**Figure 8:**
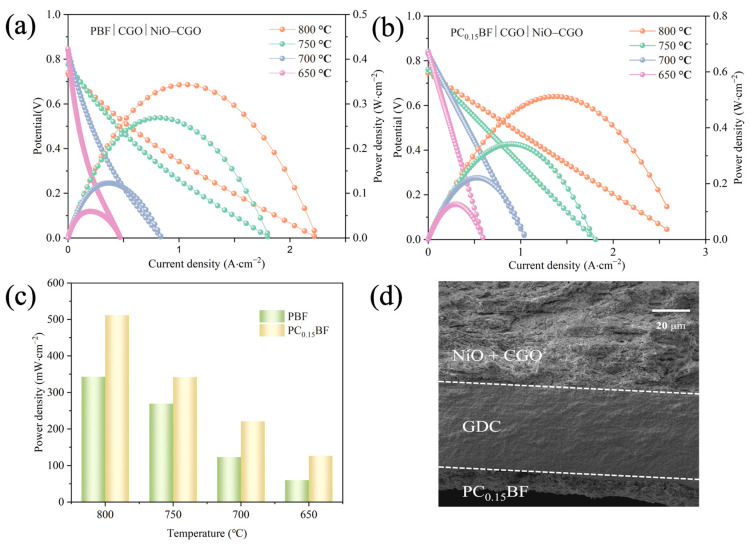
(**a**) I-V-P curve of single cell PBF|CGO|NiO+CGO; (**b**) I-V-P curve of single cell PC_0.15_BF|CGO|NiO+CGO (**c**) PPD values at different temperatures; (**d**) SEM image of PC_0.15_BF|CGO|NiO+CGO cross-section.

Figure 8a,b shows the I-V-P curve of single-cell PC_x_BF|CGO|NiO+CGO (x = 0, 0.15) when H_2_ is used as the fuel and air is used as the oxidizing agent. The current density increases with the increase in temperature. Figure 8c shows the measured peak power density (PPD) of a single cell at different temperatures. At 800 °C, the PPD value of Pr_1.85_Ca_0.15_BaFe_2_O_5+δ_ (PC_0.15_BF) exhibits excellent electrochemical performance of 512 mW·cm^−2^, which is about 49% higher than that of PBF, indicating the replacement of Pr with Ca at the A-position can improve the output power density of the battery. Figure 8d is the SEM cross-section of PC_0.15_BF|CGO|NiO+CGO single cell. No evident delamination occurs between components, indicating good chemical compatibility. However, due to Ce^4+^→Ce^3+^ in high-temperature environments, the electronic conductivity of the electrolyte increases, so the open-circuit voltage gradually decreases with the increase in temperature [38,39].

## 3. Materials and Methods

### 3.1. Powder Preparation

The powder Pr_1-x_Ca_x_BaFe_2_O_5+δ_ (x = 0, 0.05, 0.10, 0.15, 0.20) was prepared using the sol-gel method. Certain masses of Pr(NO_3_)_3_·6H_2_O, Ca(NO_3_)_2_·4H_2_O, Ba(NO_3_)_2_, and Fe(NO_3_)_3_·9H_2_O were obtained according to a stoichiometric ratio and dissolved in deionized water. This was followed by the addition of a complexing agent (EDTA and citric acid, where n_metal ion_: n_EDTA_: n_citric acid_ = 1:1: 1.5). Finally, ammonia water was added to adjust the pH value of the mixed solution to 8. A gel formed after continuous stirring at 80 °C. The obtained gel was heat-treated on the resistance furnace to obtain a fluffy black precursor. After heating the precursor at 1100 °C for 5 h (temperature rise rate 5 °C·min^−1^), the cathode powder materials Pr_1-x_Ca_x_BaFe_2_O_5+δ_ (x = 0, 0.05, 0.10, 0.15, 0.20) were obtained. The materials were named PC_x_BF (x = 0, 0.05, 0.10, 0.15, 0.20).

### 3.2. Physical and Chemical Properties

The phase composition and crystal structure of the samples were analyzed by an X-ray diffractometer (XRD, Malvern Panalytical, Empyrean, Malvern, UK) with a scanning rate of 5°/min, a scanning range of 10–80°, and Cu Kα radiation (40 kV, 40 mA, λ = 1.5418 Å). The results were refined by Rietveld using EXPGUI software(PC-GSAS). In addition, the element distribution and lattice spacing of the materials were characterized by transmission electron microscopy (TEM, JEOL, 2100F, Tokyo, Japan). Energy-dispersive X-ray and high-resolution transmission electron microscopy were performed. The surface element valence of PC_x_BF powder was analyzed using an X-ray diffractometer spectrometer (XPS, Thermo Scientific, EscaLab250Xi, Waltham, MA, USA).

The synthesized PC_x_BF powder was pressed into a rectangular strip sample at a pressure of 300 Mpa and then sintered at 1200 °C for 5 h, with a size of about 29.0 mm × 6.0 mm × 0.5 mm. The linear expansion rate of the sample was measured with a thermal dilatometer (Netzsch, DIL402C, Selb, Germany ), and the average TEC of the sample was calculated. The test was carried out in an air environment at a temperature range of 30–750 °C and a heating rate of 5 °C·min^−1^. The electrical conductivity of the material in the range of 200–800 °C was measured using an electrochemical workstation (Metrohm, PGSTAT302N, Herisau, Switzerland).

### 3.3. Preparation and Evaluation of Symmetric and Single Cells

Gd_0.1_Ce_0.9_O_1.95_ (CGO) powder was synthesized using the sol-gel method described above. After heat treatment at 800 °C for 3 h, fluffy CGO electrolyte powder was obtained. The CGO powder was placed in a circular mold with a diameter of 12 mm and pressed into a round sheet at a pressure of 200 Mpa. After holding it at 1450 °C for 5 h, a dense electrolyte sheet was obtained. The prepared cathode paste was evenly coated on both sides of the electrolyte CGO by screen printing. After drying, it was held at 1100 °C for 5 h. The symmetrical cell PC_x_BF|CGO|PC_x_BF (x = 0, 0.05, 0.10, 0.15, 0.20) required for the electrochemical impedance test was prepared. Silver paste and silver wire were used as current collectors during the test.

The pressure method was used to prepare a single anode support cell. The specific preparation process was as follows: The anode material (NiO-CGO) and the fluffy electrolyte powder were evenly spread in a circular mold with a diameter of 15 mm, dry pressed, and formed at a pressure of 200 Mpa. The pressed green half cell was held at 1450 °C for 5 h to obtain the anode support half cell. The cathode material was evenly coated on the half cell (electrolyte CGO side) using the screen printing method. After drying, the single cell was obtained by sintering it in the Muffle furnace at 1100 °C for 5 h.

The electrical conductivity of the cathode material in the range of 200–800 °C and the electrochemical impedance spectrum of the symmetric cell were tested using an electrochemical workstation. The test frequency range was 0.1–100 kHz and the AC signal amplitude was 10 mV. The output power of a single cell in the range of 650–800 °C was tested at an interval of 50 °C. In addition, scanning electron microscopy (SEM, TESCAN, GAIA3, Brno, Czech) was used to observe and analyze the morphology of the cell cross-section.

## 4. Conclusions

Pr_1-x_Ca_x_BaFe_2_O_5+δ_ was successfully prepared and used as the cathode of IT-SOFC. The results showed that Ca doping did not change the crystal structure. The Rietveld refinement results showed that with the increase in doping amount, the cell parameters gradually decreased and the lattice shrank. This material and electrolyte CGO had good chemical compatibility. The average TEC of PC_0.15_BF was higher than that of PBF. Charge compensation was achieved by increasing the average valence state of Fe or increasing the oxygen vacancy concentration, which effectively increased the adsorbed oxygen content and promoted oxygen migration and ORR at the interface of PC_x_BF|CGO. Thus, the electrochemical performance was improved and production cost was reduced. PC_0.15_BF showed the lowest *R_p_* of 0.033 Ω·cm^2^. The addition of Ca enhanced the transfer of oxygen ions in ORR. The single-cell PC_0.15_BF|CGO|NiO+CGO had the highest power density of 512 mW·cm^−2^ at 800 °C. Hence, Pr_1.85_Ca_0.15_BaFe_2_O_5+δ_ is a promising IT-SOFC cathode material.

## Figures and Tables

**Table 1:** XPS percentage values of Pr, Fe, and O elements.

Sample	Pr^3+^ (%)	Pr^4+^ (%)	Fe^3+^ (%)	Fe^4+^ (%)	(O_adsorbed_ + O_vacancy_)/O_Lattice_
x = 0.00	49.31	50.69	67.12	32.88	1.6894
x = 0.05	44.66	55.34	64.36	35.64	1.7518
x = 0.10	43.51	56.49	63.01	36.99	1.7945
x = 0.15	41.81	58.19	60.13	39.87	1.8653
x = 0.20	41.61	58.39	57.93	42.07	1.9422

## Data Availability

Data are contained within the article and Supplementary Materials.

## Supplementary Materials

The following supporting information can be downloaded at: https://www.mdpi.com/article/10.3390/molecules29132991/s1, Figure S1: (a~d): Rietveld refinement patterns of PC_x_BF samples (x = 0, 0.05, 0.1 and 0.2); FigureS2: XRD pattern of PC_0.15_BF mixed powder with CGO; Table S1: Rietveld refinement results of PC_x_BF; Table S2: Oxygen content, oxygen vacancy, average valence state of Fe^n+^
