# Supplementary material for: Ca-Doping Cobalt-Free Double Perovskite Oxide as a Cathode Material for Intermediate-Temperature Solid Oxide Fuel Cell
Source: Molecules. 2024 Jun 23;29(13):2991. doi: 10.3390/molecules29132991 (PMC11243253; doi:10.3390/molecules29132991)
Supplement: Supplementary file 1 [file molecules-29-02991-s001.zip › molecules-3046036-supplementary.pdf]

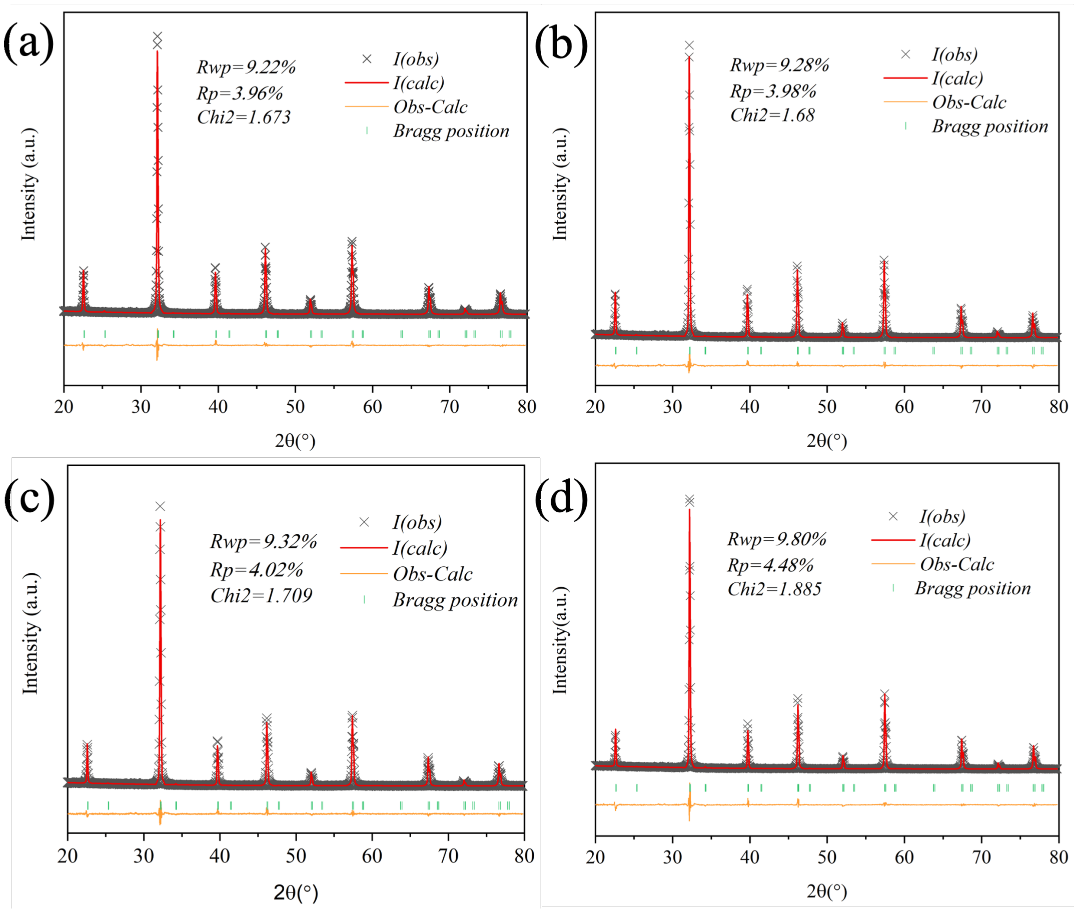

Figure S1 (a~d) Rietveld refinement patterns of PC_xBF samples (x=0, 0.05, 0.1 and 0.2)

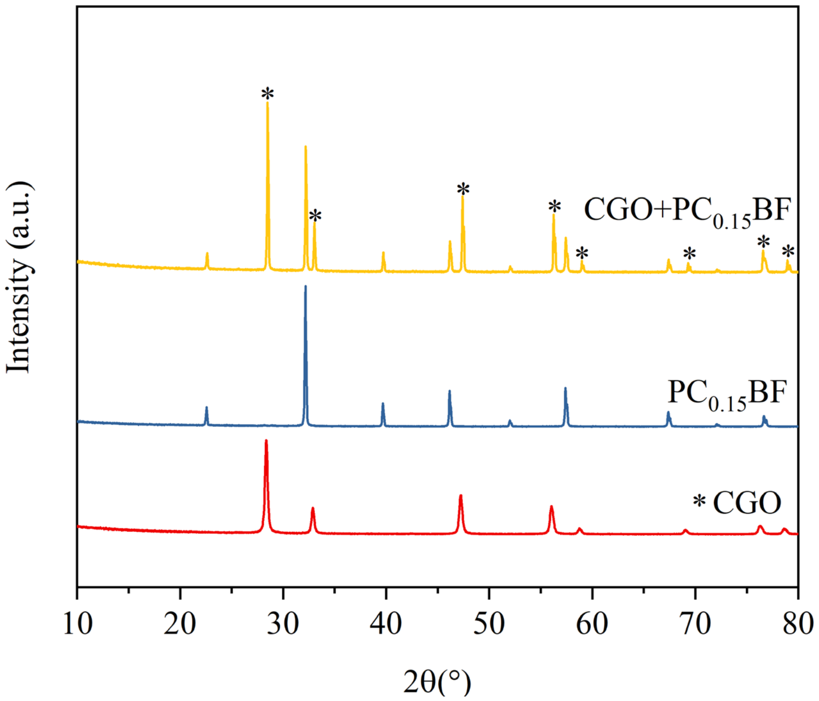

Figure S2 XRD pattern of $PC_{0.15}BF$ mixed powder with CGO

Table S1 Rietveld refinement results of PC_xBF

Sample		PBF	$PC_{0.05}BF$	$PC_{0.1}BF$	$PC_{0.15}BF$	$PC_{0.2}BF$
Space group		P4/mmm	P4/mmm	P4/mmm	P4/mmm	P4/mmm
	a=b(Å)	3.9320	3.9307	3.9293	3.9271	3.9265
Lattice	c(Å)	7.8624	7.8604	7.8572	7.8539	7.8520
parameters	V(Å^3)	121.5564	121.4408	121.3095	121.1239	121.0674
		Pr,0,0,0.5	Pr,0,0,0.5	Pr,0,0,0.5	Pr,0,0,0.5	Pr,0,0,0.5
		Occupancy=1	Occupancy=0.95	Occupancy=0.9	Occupancy=0.85	Occupancy=0.8
			Ca,0,0,0.5	Ca,0,0,0.5	Ca,0,0,0.5	Ca,0,0,0.5
	Atom		Occupancy=0.05	Occupancy=0.1	Occupancy=0.15	Occupancy=0.2
	position	Ba,0,0,0	Ba,0,0,0	Ba,0,0,0	Ba,0,0,0	Ba,0,0,0
		Occupancy=1	Occupancy=1	Occupancy=1	Occupancy=1	Occupancy=1
		Fe,0.5,0.5,0.25	Fe,0.5,0.5,0.25	Fe,0.5,0.5,0.25	Fe,0.5,0.5,0.25	Fe,0.5,0.5,0.25
		Occupancy=1	Occupancy=1	Occupancy=1	Occupancy=1	Occupancy=1
		O1,0.5,0.5,0.5	O1,0.5,0.5,0.5	O1,0.5,0.5,0.5	O1,0.5,0.5,0.5	O1,0.5,0.5,0.5
		Occupancy=1	Occupancy=1	Occupancy=1	Occupancy=1	Occupancy=1
		O2,0.5,0,0.25	O2,0.5,0,0.25	O2,0.5,0,0.25	O2,0.5,0,0.25	O2,0.5,0,0.25
		Occupancy=1	Occupancy=1	Occupancy=1	Occupancy=1	Occupancy=1
		O3,0.5,0.5,0	O3,0.5,0.5,0	O3,0.5,0.5,0	O3,0.5,0.5,0	O3,0.5,0.5,0
		Occupancy=1	Occupancy=1	Occupancy=1	Occupancy=1	Occupancy=1
Refinement	R_p	3.96%	3.98%	4.02%	4.12%	4.48%
Parameters	R_{wp}	9.22%	9.28%	9.32%	9.85%	9.8%
	χ^2	1.673	1.68	1.709	1.774	1.885

Quantitative analysis of oxygen nonstoichiometry in PC_xBF by iodometry titration

The oxygen content of the material $Pr_{1-x}Ca_xBaFe_2O5_{+\delta}$ (x=0, 0.05, 0.1, 0.15, 0.2, PC_xBF) is measured using iodine titration, the corresponding principle is as follows, assuming that the valence of Fe in the PC_xBF material is 1+p. A sample of mass m_1 is placed into an iodine measuring flask, and an excess of KI and a dilute HCl solution are added, and the following chemical reaction occurs in the flask:

$$Fe^{1+p}+(p-1)I^- \rightarrow \frac{p-1}{2}I_2+Fe^{2+} \qquad \text{Equation（S1）}$$

Then use the calibrated $Na_2S_2O_3$ solution to titrate the I_2 in the solution

$$I_2+2Na_2S_2O_3 \rightarrow 2NaI+Na_2S_4O_6 \qquad \text{Equation（S2）}$$

V_1, C and M represent the volume of $Na_2S_2O_3$ solution consumed in the experiment, the concentration of $Na_2S_2O_3$ solution and the molar mass of the sample, respectively, according to Eq. S2:

$$CV_1(p-1)=2\frac{m_1}{M} \qquad \text{Equation（S3）}$$

According to the measured data to calculate the average valence of Fe ions. Known valence of other metal ions, based on the principle of conservation of charge, the compound in the positive and negative valence is ultimately equal to zero, through the titration results can be calculated to find the size of the P-value, so that you can find out the oxygen content:

$$5+\delta=2.5-0.5x+(1+P) \qquad \text{Equation（S4）}$$

where x is the amount of Ca^{2+} doping.

Table S2 Oxygen content, oxygen vacancy, average valence state of Fe^{n+}

PC_xBF	5+δ	Oxygen vacancy	Fe^{n+} average valance state
x=0.00	5.80	0.20	3.3
x=0.05	5.79	0.21	3.315
x=0.10	5.78	0.22	3.33
x=0.15	5.77	0.23	3.345
x=0.20	5.76	0.24	3.35
